# Consensus-Based Pharmacophore Mapping for New Set of *N*-(disubstituted-phenyl)-3-hydroxyl-naphthalene-2-carboxamides

**DOI:** 10.3390/ijms21186583

**Published:** 2020-09-09

**Authors:** Andrzej Bak, Jiri Kos, Hana Michnova, Tomas Gonec, Sarka Pospisilova, Violetta Kozik, Alois Cizek, Adam Smolinski, Josef Jampilek

**Affiliations:** 1 Department of Chemistry, University of Silesia, Szkolna 9, 40007 Katowice, Poland; violetta.kozik@us.edu.pl; 2 Division of Biologically Active Complexes and Molecular Magnets, Regional Centre of Advanced Technologies and Materials, Faculty of Science, Palacky University, Slechtitelu 27, 78371 Olomouc, Czech Republic; michnova.hana@gmail.com (H.M.); sharka.pospisilova@gmail.com (S.P.); josef.jampilek@gmail.com (J.J.); 3 Department of Chemical Drugs, Faculty of Pharmacy, Masaryk University, Palackeho 1, 60200 Brno, Czech Republic; t.gonec@seznam.cz; 4 Department of Infectious Diseases and Microbiology, Faculty of Veterinary Medicine, University of Veterinary and Pharmaceutical Sciences, Palackeho 1946/1, 61242 Brno, Czech Republic; cizeka@vfu.cz; 5 Central Mining Institute, Pl. Gwarków 1, 40-166 Katowice, Poland; smolin@gig.katowice.pl

**Keywords:** hydroxynaphthalenecarboxamides, lipophilicity, antistaphylococcal activity, antitubercular activity, MIC, MTT assay, CoMSA, IVE-PLS, similarity-activity landscape index

## Abstract

A series of twenty-two novel *N*-(disubstituted-phenyl)-3-hydroxynaphthalene- 2-carboxamide derivatives was synthesized and characterized as potential antimicrobial agents. *N*-[3,5-bis(trifluoromethyl)phenyl]- and *N*-[2-chloro-5-(trifluoromethyl)phenyl]-3-hydroxy- naphthalene-2-carboxamide showed submicromolar (MICs 0.16–0.68 µM) activity against methicillin-resistant *Staphylococcus aureus* isolates. *N*-[3,5-bis(trifluoromethyl)phenyl]- and *N*-[4-bromo-3-(trifluoromethyl)phenyl]-3-hydroxynaphthalene-2-carboxamide revealed activity against *M. tuberculosis* (both MICs 10 µM) comparable with that of rifampicin. Synergistic activity was observed for the combinations of ciprofloxacin with *N*-[4-bromo-3-(trifluoromethyl)phenyl]- and *N*-(4-bromo-3-fluorophenyl)-3-hydroxynaphthalene-2-carboxamides against MRSA SA 630 isolate. The similarity-related property space assessment for the congeneric series of structurally related carboxamide derivatives was performed using the principal component analysis. Interestingly, different distribution of mono-halogenated carboxamide derivatives with the –CF_3_ substituent is accompanied by the increased activity profile. A symmetric matrix of Tanimoto coefficients indicated the structural dissimilarities of dichloro- and dimetoxy-substituted isomers from the remaining ones. Moreover, the quantitative sampling of similarity-related activity landscape provided a subtle picture of favorable and disallowed structural modifications that are valid for determining activity cliffs. Finally, the advanced method of neural network quantitative SAR was engaged to illustrate the key 3D steric/electronic/lipophilic features of the ligand-site composition by the systematic probing of the functional group.

## 1. Introduction

The comprehensive specification of the target-ligand interaction content in the rational drug design is a computationally intense issue that requires at least four German G’s: Geschick (skill), Geduld (patience), Geld (money), and Glück (luck) [1]. Hence, the decision-making process of hit identification→lead generation→lead optimization→candidate nomination in the synthetic efforts during drug discovery ought to be supported by a variety of advanced in silico approaches. On the whole, the computer-assisted manipulation of host-quest structures can be dichotomized into direct target-dependent (structure-based) as well as indirect drug-related (ligand-based) methodologies [2]. Unfortunately, there is no a priori guideline based on which a procedure performs best in the search for promising drug molecule for a given disease; therefore, the integrated (collaborative) approach is advisable to advocate chemical intuition [3]. Whenever no structure or model of the target molecule is accessible at the atomic level the pharmacophore-guided concept can be employed for mapping of the binding/active site [4]. The inspiration for pharmacophoric pattern study stems loosely from the intermolecular recognition concept assuming that interchangeable groups that are similar in size, shape, or electronic distribution are likely to induce similar effects on binding affinities (neighbor behavior) [5]. Conceptually, the straightforward tenet of substituent interchangeability and complementarity inherently favors the similarity principle, especially in structure-activity (SAR) modeling, where congeneric series of molecules should exhibit similar pharmacological profile [6]. In this context, validation is one of those words… that is constantly used and seldom defined as was stated by Feinstein [7]. Obviously, a comprehensive mapping of the topology and/or topography of a compound into the property-based chemical space (CS) is not feasible in principal as was noticed by an anonymous reviewer, since it is estimated that up to 10^100^ structures can be synthesized with potentially 10^60^ small molecules of less than 30 non-hydrogen atoms and up to 10^40^ pharmacologically relevant compounds [8]. Hence, only a limited exploration of the factual chemical space (FCS) is achievable (10^8^), mainly employing structurally-related structures [9,10]. On the other hand, the clever management of pharmacophore-related information can provide hints that might be incorporated at the synthesis stage.

Basically, the pseudoreceptor mapping of pharmacophoric features can be classified into similarity-driven and hypothesis-related approaches, respectively [11]. Roughly speaking, the hypothesis-based procedures employ frequently machine learning techniques conjugated with variable selection/weighting algorithms to prioritize robust linear or non-linear SAR models [12]. The quantitative comparison of ligand surfaces using electrostatic potential maps (CoMSA) and field-based descriptors (CoMFA) can provide a more realistic picture of mutual drug-receptor recognition phenomena; however, it is only a crude approximation of the underlying biological reality [13,14]. On the other hand, the similarity principle is still questionable since similarity depends strongly on both descriptor and/or similarity score used. Despite some drawbacks, distance-related similarity searching with a numerical measure of the intermolecular resemblance between two objects, each described by a number of attributes, contributes favorably in SAR practice [15]. Obviously, certain molecular signatures such as lipophilicity can correlate with bioavailability, therefore in silico filters are meaningful in restricting the values of structural or physicochemical descriptors to ADMET-friendly property space [16]. Estimation of the sustainable equilibrium between ADMET-related properties and desirable drug affinity to the receptor can be illustrated graphically by enhancement of the planar (2D) similarity-driven projection with activity data to form a biological response surface [17]. Intuitively, SAR landscapes are not homogenous in nature, but rather heterogeneously distributed regions resembling more rugged topography of Poland’s Tatra Mountains than the gently rolling Flint Hills of Kansas [18]. Chemically, smooth and flat regions of SAR landscape indicate areas where subtle structural changes are accompanied by tiny variations in biological response according to the similarity principle. Conversely, sharp and non-uniform areas called activity cliffs highlight structurally-related compounds that are characterized by exorbitant changes in potency to target (magic methyl phenomenon) [19]. From the drug hunter’s perspective, successful exploration of jagged landscape parts provides a guideline to a non-trivial question about the structural modifications that boost or demolish the biological activity. In fact, a range of similarity metrics and fingerprint representation is employed to numerically quantify the SAR smoothness, for instance, the structure-activity landscape index (SALI) [20].

Hydroxynaphthalenecarboxanilide scaffolds make it possible to design a wide variety of compounds, especially with anti-infective [21,22,23,24,25,26] and antineoplastic activity [27,28]. 3-Hydroxynaphthalene-2-carboxanilides (which are direct radical analogs of salicylanilides) were investigated as well [29,30]. The activity of these structurally simple compounds is dependent on the mutual position of the carboxamide and the phenolic moieties [21,22,23,24,25,26,27,28,31,32]. In other words, various substitutions of the crucial phenolic motif determine a spectrum of different biological activities. Roughly speaking, the carboxamide fragment that mimics a peptide bond seems to be pivotal for the binding affinity, since a compound is bound to its targets using this molecular anchor. One can assume that the anilide part of the molecule has a direct impact also on the physicochemical properties and binding strength of the tested compounds to the potential target. As a matter of fact, hydroxynaphthalenecarboxanilide analogs can be classified as multi-target compounds, since they are able to affect various target structures, especially in microbial pathogens (similarly to salicylanilides) [33]. These types of compounds are able to inhibit glucose uptake, oxidative phosphorylation, two-component regulatory systems of bacteria, interleukin-12p40 production, various bacterial enzymes, respectively. Moreover, they can bind to protein kinase epidermal growth factor receptor, destruct the cellular proton gradient as proton shuttles, etc. [34]. Thus, based on the experience with various substituents on the aniline core a series of novel 22 *N*-(disubstituted-phenyl)-3-hydroxynaphthalene-2-carboxamides, was synthesized and in vitro tested against several microbial strains [26,35,36,37]. The similarity related property space assessment for the set of carboxamide derivatives was performed using the principal component analysis (PCA) [38,39]. Moreover, the quantitative sampling of similarity related activity landscape provided a subtle picture of favorable and disallowed structural modifications that are valid for determining the activity cliffs [40]. Finally, the complex approach of the machine learning using neural network was employed in quantitative SAR to specify the potentially valid 3-dimensional steric/electronic/ lipophilic features of the ligand-receptor composition by the systematic sampling of the functional group resulting in the production of an averaged selection-driven pharmacophore pattern [26].

## 2. Results and Discussion

### 2.1. Synthesis and Physicochemical Properties

**Scheme 1:**
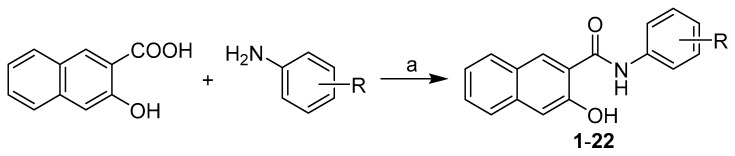
Synthesis of *N*-(disubstituted-phenyl)-3-hydroxynaphthalene-2-carboxamides **1**–**22**. Reagents and conditions: (a) PCl_3_, chlorobenzene, microwave reactor, 45 min [31].

All studied *N*-(disubstituted-phenyl)-3-hydroxynaphthalene-2-carboxamides were prepared according to Scheme 1 as described previously by Kos et al. [31]. The condensation of 3-hydroxynaphthalene-2-carboxylic acid with appropriate disubstituted anilines using phosphorus trichloride in dry chlorobenzene under microwave conditions gave a series of target 3-hydroxy-*N*-arylnaphthalene-2-carboxanilides **1**–**22** (see Table 1).

### 2.2. In Vitro Antimicrobial Activity

The biological screening of all the compounds was performed against the reference and quality control strain *Staphylococcus aureus* ATCC 29213, three clinical isolates of methicillin-resistant *S. aureus* (MRSA) [41], and against *Mycobacterium tuberculosis* H37Ra ATCC 25177. The efficiency of the compounds was expressed as the minimum inhibitory concentrations (MICs), that are defined for bacteria as 90% (IC_90_) reduction of growth in comparison with the control (see Table 1). The MIC (IC_90_) value is routinely and widely used in bacterial assays as a standard detection limit according to the Clinical and Laboratory Standards Institute (CLSI) [42,43]. Based on the range of observed MIC values, it can be summarized that compounds expressed a wide range of potencies. Some of them, especially substituted by a trifluoromethyl moiety were much more active than standards, clinically used drugs. *N*-[3,5-bis(Trifluoromethyl)phenyl]- (**13**) and *N*-[2-chloro-5-(trifluoromethyl)- phenyl]-3-hydroxynaphthalene-2-carboxamide (**20**) showed submicromolar (MICs 0.16–0.68 µM) antistaphylococcal activity; and compounds **16**–**19** and **21** demonstrated antistaphylococcal activity ranging from 2.4 to 11.5 µM. As MICs against MRSA isolates were, in fact, comparable with the MIC values observed against methicillin-susceptible *S. aureus* ATCC 29213, it could be assumed that the presence of *mecA* gene did not affect the activity of these compounds [44].

The same compounds, i.e., **13**, *N*-[4-bromo-3-(trifluoromethyl)phenyl]-3-hydroxy-naphthalene-2-carboxamide (**16**), **12**, and **17**–**21** showed activity against *M. tuberculosis* ranging from 9.75 to 12.0 µM and comparable with that of rifampicin. Therefore, a standard MTT (3-(4,5-dimethylthiazol-2-yl)-2,5-diphenyltetrazolium bromide) assay was performed with the chosen most effective compounds against *M. tuberculosis* H37Ra. The MTT test can be used to assess cell growth by measuring respiration. The MTT measured viability of mycobacterial cells less than 70% after exposure to the MIC values for each tested compound is considered as a positive result of this assay. This low level of cell viability indicates inhibition of cell growth by inhibition of respiration [45,46]. It can be concluded that compounds **12** (R = 3,5-Cl, 27%), **13** (R = 3,5-CF_3_, 6%), **19** (R = 5-CF_3_-3-F, 41%), **20** (R = 5-CF_3_-2-Cl, 5%), and **21** (R = 4-CF_3_-3-F, 46%) showed the viability of *M. tuberculosis* that was significantly less than 70% at the tested concentration equal to MICs [47].

Based on the above findings, it can be assumed that one mechanisms of actions of these so-called multi-target compounds appears to be the inhibition of bacterial respiration by the inhibition of ATP synthase (F_0_F_1_ complex) or cytochrome bc1 [21,22,23,24,25,26,48]. In addition, the structurally-related molecules can destroy the cellular proton gradient as proton shuttles that is evidenced by their ability to inhibit photosynthetic electron transport (PET) in chloroplasts [29,30,31,49,50,51]. In fact, a good correlation was found between PET inhibition and the antibacterial/antimycobacterial activities [52,53]). It seems that other possible sites of action in bacterial cells are possible; therefore, further studies at the molecular level are needed.

### 2.3. Molecular Similarity Assessment

**Figure 1:**
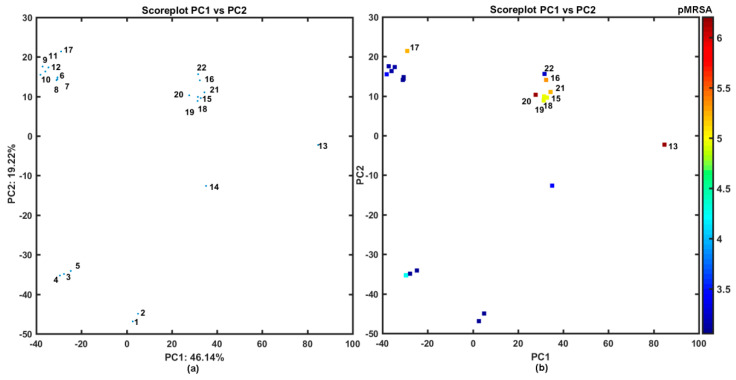
Projection of carboxamides **1**–**22** on plane defined by first vs. second principal components for Dragon descriptors (**a**) with MRSA activity in logarithmic scale (**b**) colors code numerical values of pMRSA activity. Molecules with pMRSA > 4 are numerically labeled.

The similarity-guided evaluation of the activity profile for the congeneric series of structurally-related *N*-(disubstituted-phenyl)-3-hydroxynaphthalene-2-carboxamide derivatives was performed using the principal component analysis (PCA) on the pool of 2762 descriptors generated by Dragon 6.0 software, where constant or nearly constant descriptors (standard deviation < 0.0001) were *a priori* excluded (overall 2123 variables) [54]. The multi-dimensional data were arranged in X_22×2762_ matrix with columns and rows representing variables and molecules, respectively. The centered and standardized data were subsequently subjected to PCA, where compression efficiency is directly related to the quantity of uncorrelated variables. The high percentage of total variance described by the first three principal components (72.69%) suggests that variables are highly inter-correlated. The first two PCs characterized 65.36% of the overall variance; therefore, the projection of the selected properties on the plane defined by the first two PCs with respect to carboxamide chemotype was conducted as illustrated in Figure 1.

Despite the fact, that carboxamide derivatives **1**–**22** have a common chemotype based on hydroxynaphthalene ring, peptide-like linker, and phenyl substituent, mainly two heterogeneous structural subfamilies can be formed along the PC1: the first one (PC1 < 0) with compounds **3**–**12**, **17** and the second group (PC1 > 0) containing the remaining ones (see Figure 1a). It is noticeable that very active compound **13** occupies a distinct location of the PC1 vs. PC2 plane, while fairly potent compounds group together (20 < PC1 < 40), as shown in Figure 1b. Interestingly, different distribution of mono-halogenated carboxamide derivatives with the –CF_3_ substituent (molecules **15**, **16**, **18**–**22**) is accompanied by the increased activity profile (see Figure 1b).

**Figure 2:**
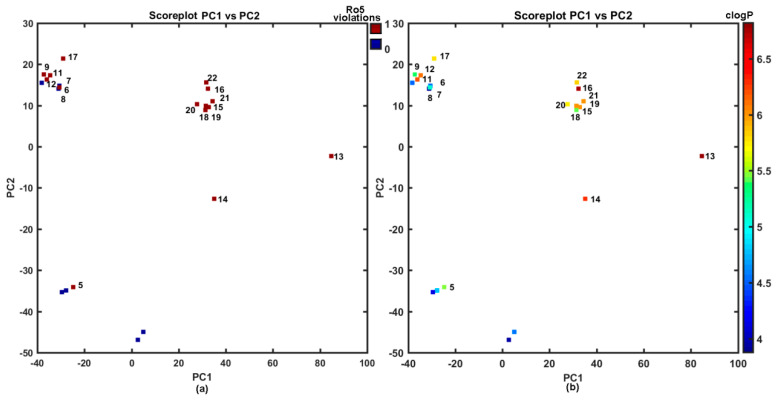
Projection of carboxamides **1**–**22** on the plane defined by first vs. second principal components for Dragon descriptors with Ro5 rule violations (**a**) and calculated lipophilicity clogP (**b**) colors code numerical values of clogP and Ro5 violations. Molecules with Ro5 violation and clogP > 5 are numerically labeled.

In fact, the majority of the investigated carboxamides do not strictly obey the Lipinski’s Rule of Five (Ro5), where the set of threshold values was imposed on the specific molecular descriptors (MW ≤ 500, HBD ≤ 5, HBA ≤ 10, clogP ≤ 5) to specify drug-like property space (see Figure 2a); however, a good drug-like score does not make a molecule a drug and vice versa [55,56,57]. On the other hand, the violations of any two of the Ro5 conditions reduces the probability of a compound to be orally bioavailable as suggested anonymous Reviewer. Clearly, ADMET-friendly properties such as lipophilicity are meaningful in the context of specific ligand-receptor interactions; therefore, the lipophilic profile for the analyzed set of compounds is displayed in Figure 2b.

**Figure 3:**
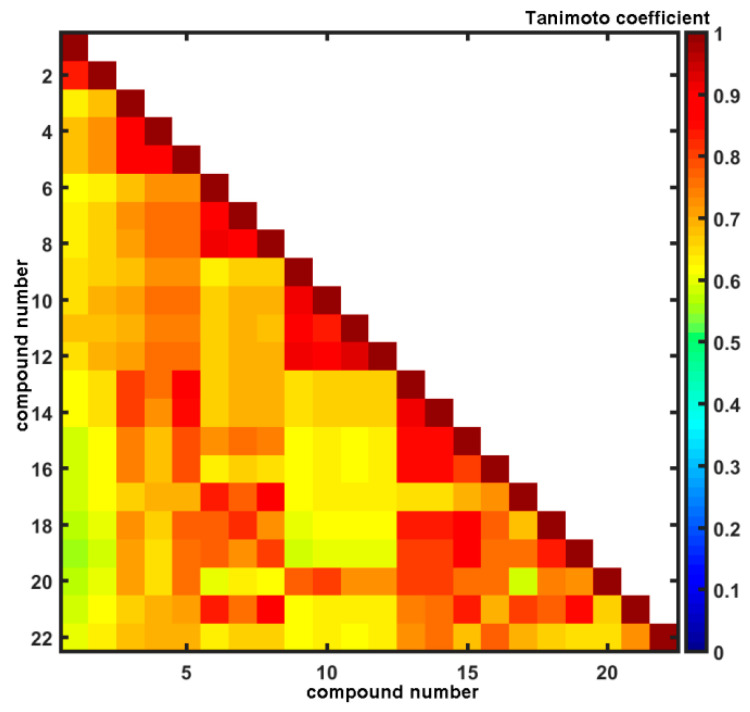
Triangular matrix of Tanimoto coefficients for **1**–**22** carboxamides.

Unarguably, the core of many SAR-based methods is the similarity principle in the structural space assuming that similar compounds are expected to display similar physicochemical and biological properties. Conceptually, medicinal chemists have adopted a simplified view that similarity between two objects can be quantitatively expressed by the function of common features [58]. Despite obvious oversimplification of the similarity quantification (e.g., some similarity scores exhibit size-dependent behavior), it is still a powerful concept, where the number of molecular characteristics might be denoted by a bit-string representation (sometimes augmented with the scaling coefficients). The pair-wise descriptor-based structural resemblance/relatedness can be numerically evaluated by a variety of relative distance metrics (Hamming or Euclidean measures) or absolute comparison using Tanimoto coefficient calculated for OpenBabel fingerprints. The distribution of Tanimoto coefficients for the analyzed set of compounds was investigated revealing that the greatest frequency was recorded in the range of 0.65 < T < 0.70, respectively. A triangular matrix of T_22×22_ in Figure 3 indicates the structural dissimilarities of dichloro-substituted (compounds **9**–**12**) and dimetoxy-substituted (compounds **1**, **2**) isomers from the remaining ones that confirm our previous PCA findings (see Figure 1a). On the other hand, in the Tanimoto matrix compound **13** is high similar to compounds **14**–**22**, but PCA showed a different result as indicated by an anonymous reviewer. It should be noted that the PCA-based similarity analysis is performed in the reduced PC1 vs. PC2 space, that describes 65% of the overall variance.

**Figure 4:**
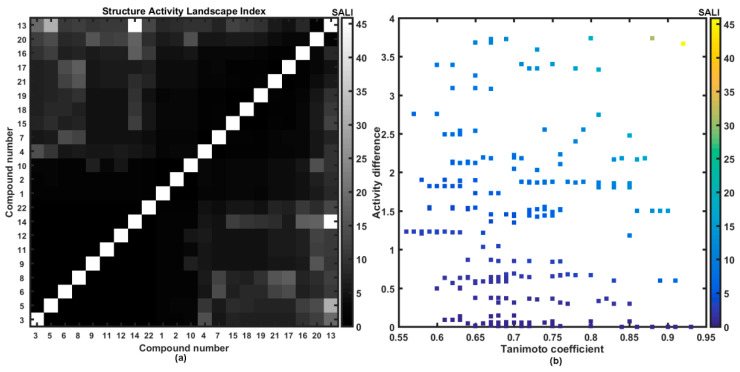
Grayscaled SALI plot with compounds ordered with increasing ATCC 29213 activities (**a**) and neighboring plot (**b**) for **1**–**22** carboxamides.

The 2D image of molecular similarity augmented with biological affinity profile provides a coherent visualization tool for the systematic illumination of SAR trends (continuity areas and/or activity cliffs) in the form of the structure-activity landscape indexes (SALI) [13,14,15,16]. The greater density of compounds that cover the factual chemical space (FCS) the more accurate specification of activity cliffs; however even relatively sparse sampling of FCS can be sufficient to construct a reliable activity landscape. As a matter of fact, the detection of sharply non-uniform regions that form steep cliffs in the activity landscape depends critically on the availability of structurally-related compounds with discernible variations in the biological response data. It is obvious that for closely related molecules (e.g., stereoisomers where T→1) SALI→infinity; therefore, such values were substituted by the largest SALI value. Figure 4a presents the symmetrical grayscaled heatmap of SALI for the investigated set of carboxamides, where axes correspond to a molecule number sorted according to increasing affinity ATCC 29213 (ΔpATCC = 3.75) with a legend indicating the range of SALI values. In fact, two types of spots can be noticed on the heatmap: the white representing the highest numerical SALI values and the black that indicate minimal ones, respectively.

Located at the right lower part of the SALI plane (or symmetrically positioned in the upper left part) are pairs of molecules that potentially form the activity cliff that is formally manifested via demolition of activity for similar structures (lighter spots in Figure 4a). Interestingly, the substitution of hydrogen atoms in methyl groups of molecule **5** with fluorine resulted in the boost of potency observed for molecule **13**. Moreover, the potent molecule **13** is accompanied by inactive molecule **14**, where one trifluoromethyl group was isomerically (*meta*→*orto*) replaced with one methyl substituent. The mentioned structural modifications, that unfavorably affect the affinity profile, can be tracked down on the neighborhood plot (see Figure 4b), where the structurally-related pairs of molecules (T > 0.85) are plotted versus differences in the biological activity and color-coded by higher SALI values as well. It seems, that further dense sampling of rough regions (the upper right corner) in the numerical SALI plane (T > 0.75 and pIC_50_ > 2) is necessary to specify the valid SAR boundaries for the investigated series of carboxamides.

### 2.4. Probability-Driven Pharmacophore Mapping

**Figure 5:**
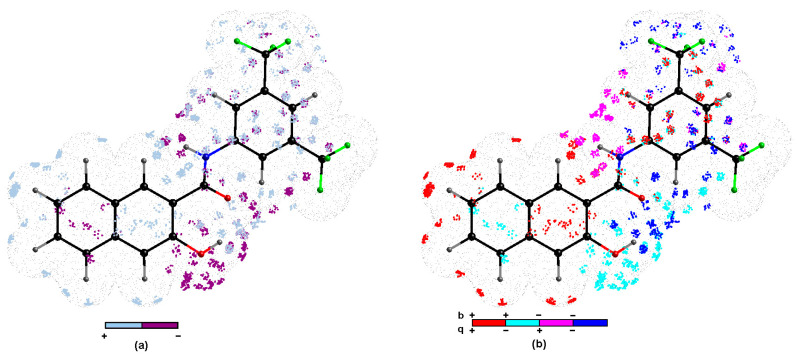
Spatial sectors with largest contribution into MRSA potency selected by CoMSA IVE-PLS for **14**/**8** training/test set samplings. Colors code their contribution (**a**) and four possible combination of mean charge q and correlation coefficient b values (**b**) **1**–**22** carboxamides. Reference compound **13** was illustrated as the most potent molecule.

In order to explore the key 3D steric/electronic/lipophilic features of the ligand-site composition the systematic probing of the functional group complementarity inevitably led to the introduction of pharmacophore concept in SAR studies [59]. In the simplest case, the relative arrangement of pharmacophoric properties is specified for the homogenous subfamily of compounds that share the common structural scaffold (chemotype). In reality, drug hunters aspire to enhance the SAR applicability domain for non-congeneric series of molecules, but accurate de novo prediction of modeled property for the entire universe of chemicals (CS) is still an elusive and enigmatic operation. Moreover, the model robustness and predictive power are strongly dependent on the training/test subset division, but no correlation between good retrospective performance and good prospective performance was observed (Kubinyi paradox) [60,61]. In fact, there are no specific rules to place particular molecules into the training/test groups; therefore, multiple and interchangeable training/test assignment was proposed for the probability-oriented pharmacophore mapping stemming from stochastic model validation (SMV) approach [3,5,13,57]. Fortunately, the CPU-intense PLS calculations were viable for the entire pool of systematically generated training/test populations ($C_{22}^{8}$ ≈ 3 × 10^5^) for CoMSA MRSA 63718 modeling. The frequency distribution of test compounds for models with preferable $q_{cv}^{2}$ ≥ 0.5 and $q_{test}^{2}$ ≥ 0.5 parameters (more efficient than flipping a coin) revealed that active (**17** and **20**) and inactive (**9** and **22**) molecules were noticeably over-represented. The preferential over-representation of some compounds in the test set suggests the possibility of optional (or random) generation of better models; therefore, the investigation of all possible training/test set combinations seems advisable. In other words, better models were recorded for training sets depleted of the indicated molecules. Additionally, an averaged selection-driven pharmacophore pattern was produced based on the regions of the pretty high model ability and predictability according to the IVE-PLS (iterative variable elimination partial least squares) procedure described elsewhere [62]. A listing of spatial zones with favorable and unfavorable contributions of CoMSA descriptors is provided in Figure 5.

The impact of the surface/charge descriptors on the ligand-site affinity is indicated by the regions of positive and negative potency contribution (Figure 5a) and four combinations of charge values versus the mean regression coefficients (Figure 5b), respectively. It is not straightforward to translate pharmacophore-related points of the space into the corresponding pseudoreceptor model with privileged zones, that hypothetically harbors putative drug molecule, while the bundle of steric and electrostatic features was specified on the averaged surface of the receptor-selective ligand molecules. The three-dimensional distribution plot shown in Figure 5a demonstrates the contribution of hydroxynaphthalene ring, peptide-like linker, and phenyl group. Oddly enough, the negatively charged surface areas of the hydroxyl substituent contribute unfavorably, mainly due to steric and/or electronic factors, while the gray 3D polyhedrals surrounding carbonyl groups depict the spatial areas that were foreseen as favorable (see Figure 5a,b). Noticeably, the dominant gray spheres in the close proximity of position 3 in the phenyl group (Figure 5a) correspond well with the increased electron density on fluorine atoms of the –CF_3_ substituent, that confirms the tendency recorded for carboxamide derivative, where activity profile for meta substitution can be basically ranked as trifluoromethyl >> methyl. Similarly, the negatively charged substituent directly attached to the phenyl group at position 5 contributes favorably to carboxamides binding affinity as suggested by dark blue zones in Figure 5a and data recorded in Table 1, respectively. In fact, the interpretation of an average pharmacophore pattern is common for a set of congeneric subfamilies, and provides subtle hints useful at the stage of structure-related activity modifications. In fact, no particular pharmacophore interaction was indicated as dominant; however, the pharmacophore visualization based on the consensus 3D QSAR modeling provides the spatial map of chemical groups/atoms potentially relevant for increasing/decreasing the activity profile of the analyzed molecules.

### 2.5. Advanced Antimicrobial Evaluation

#### 2.5.1. Combined Effect

The most potent antistaphylococcal compounds **13**, **16**, **17**, **20**, and **21** were studied in combination with ciprofloxacin to evaluate their potential additive effect on the resistant isolate MRSA SA 630 of a given antibacterial chemotherapeutic. The method of minimal fractional inhibitory concentration index (FICI) in a microtitration plate was used. FICI ≤ 0.5 means synergy; 0.5 < FICI < 1 means additivity; 1 ≤ FICI < 4 means indifference; and FICI ≥ 4 means antagonism, respectively [63,64]. All the results are included in Table 2.

Synergistic activity was observed for the combinations of compounds **16** and **17** with ciprofloxacin (CPX) against MRSA SA 630. The combination of compound **21** with CPX showed additivity against this isolate as well. Combinations of all tested compounds with oxacillin (OXA) were indifferent. This indifference shows no interference of the tested compounds with *mec*A gene and can exclude interaction with the cell wall. The mechanism of resistance to CPX in MRSA SA 630 has not yet been precisely described. Generally, the common mechanism of resistance to fluoroquinolones is active efflux; so, a possible explanation of synergy may be inhibition of efflux pumps.

#### 2.5.2. Time-Kill Assay

**Figure 6:**
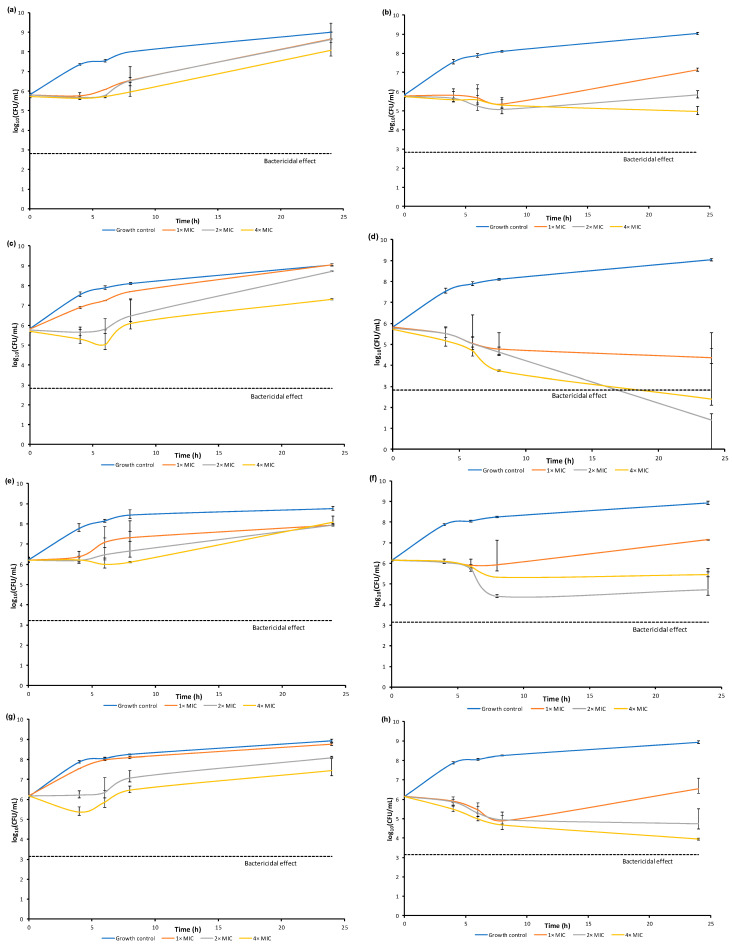
Comparison of dynamics of antibacterial activity of selected compounds **16**, **17**, **20**, **21** against *S. aureus* ATCC 29213 (**a**–**d**) and MRSA SA 3202 (**e**–**h**).

Compounds **16**, **17**, **20**, and **21** were chosen for the evaluation of the dynamics of the antibacterial activity by the time-kill curves method against *S. aureus* ATCC 29213 and MRSA SA 3202 (Figure 6). Compound **13** was not included in this experiment by reason of too low MIC, which is limiting for this methodology. The activity was tested at concentrations equal to 1×MIC, 2×MIC, and 4×MIC at timepoints 0, 4, 6, 8, and 24 h from the beginning of incubation.

Most of the tested compounds had a bacteriostatic effect. The activity of the compound **16** depended on the concentration; the static effect was observed at concentration 4×MIC at 4 and 6 h after the start of incubation for *S. aureus* ATCC 29213 and at 4, 6, and 8 h for MRSA SA 3202. The increase in the amount of CFU at 24 h could be caused by the selection of resistant mutants. Compound **17** showed the highest effect at 8 h for both tested strains. In the case of *S. aureus* ATCC 29213, there is only a small difference between concentrations at that timepoint. On the other hand, the most active concentration against MRSA SA 3202 was 2×MIC. Compound **20** showed concentration dependence with the highest effect at 4×MIC at 6 h for *S. aureus* ATCC 29213 and 4 h for MRSA SA 3202. Bactericidal effect (≥log_10_3 decrease) was observed only in the case of compound **21** at concentrations 2×MIC and 4×MIC at 24 h of incubation against *S. aureus* ATCC 29213. All the tested concentrations of this compound showed at least 90% killing at 8 and 24 h of incubation against this strain. In the case of MRSA SA 3202, 90% killing at all the three concentrations at 8 h of incubation was also observed; the concentration 4×MIC at 24 h timepoint showed actually 99% killing. Both strains showed time-dependent killing.

## 3. Materials and Methods

### 3.1. General Methods

All reagents were purchased from Merck (Sigma-Aldrich, St. Louis, MO, USA) and Alfa (Alfa-Aesar, Ward Hill, MA, USA). Reactions were conducted using a CEM Discover SP microwave reactor (CEM, Matthews, NC, USA). The melting points were specified using a Kofler hot-plate apparatus HMK (Franz Kustner Nacht KG, Dresden, Germany) and are maintained uncorrected. Infrared (IR) spectra were recorded with Smart MIRacle™ ATR ZnSe for Nicolet™ Impact 410 Fourier-transform IR spectrometer (Thermo Scientific, West Palm Beach, FL, USA). The spectra were received by the accumulation of 256 scans with 2 cm^−1^ resolution in the region of 4000–650 cm^−1^. All ^1^H- and ^13^C-NMR spectra were recorded using an Agilent VNMRS 600 MHz system (600 MHz for ^1^H and 150 MHz for ^13^C; Agilent Technologies, Santa Clara, CA, USA) in dimethyl sulfoxide-*d*_6_ (DMSO-*d*_6_). ^1^H and ^13^C chemical shifts (δ) are reported in ppm. High-resolution mass spectra were measured using a high-performance liquid chromatograph Dionex UltiMate^®^ 3000 (Thermo Scientific) coupled with an LTQ Orbitrap XL™ Hybrid Ion Trap-Orbitrap Fourier Transform Mass Spectrometer (Thermo Scientific) equipped with a HESI II (heated electrospray ionization) source in the positive mode.

### 3.2. Chemistry

#### General Procedure for Synthesis of Carboxamide Derivatives **1**–**22**

3-Hydroxynaphtalene-2-carboxylic acid (1.0 g, 5.3 mM) was suspended in dry chlorobenzene (30 mL) at ambient temperature and phosphorus trichloride (0.23 mL, 2.7 mM, 0.5 eq.), and the corresponding substituted aniline (5.3 mM, 1 eq.) was added dropwise. The reaction mixture was transferred to the microwave reactor, where the synthesis was conducted (1st phase: 10 min, 100 °C, 100 W; 2nd phase: 15 min, 120 °C, 500 W; 3rd phase: 20 min, 130 °C, 500 W). Subsequently, the mixture was cooled to 60 °C, and hence the solvent was removed to dryness under reduced pressure. The residue was washed with hydrochloric acid and water. The crude product was recrystallized from EtOH.

*N-(2,5-Dimethoxyphenyl)-3-hydroxynaphthalene-2-carboxamide* (**1**). Yield 57%; Mp 186–188 °C; IR (cm^−1^): 3425, 2934, 2836, 1604, 1592, 1538, 1490, 1445, 1434, 1280, 1217, 1183, 1144, 1050, 1024, 872, 862, 785, 751, 737, 711; ^1^H-NMR (DMSO-*d*_6_), δ: 11.79 (s, 1H), 11.13 (s, 1H), 8.70 (s, 1H), 8.24 (s, 1H), 7.98 (d, 1H, *J* = 8.1 Hz), 7.78 (d, 1H, *J* = 8.1 Hz), 7.52 (t, 1H, *J* = 7.1 Hz), 7.37 (t, 1H, *J* = 7.3 Hz), 7.36 (s, 1H), 7.03 (dd, 1H, *J* = 9.2 Hz, *J* = 1.5 Hz), 6.66 (ddd, 1H, *J* = 9.2 Hz, *J* = 7.7 Hz, *J* = 1.5 Hz), 3.87 (s, 3H), 3.74 (s, 3H); ^13^C-NMR (DMSO-*d*_6_), δ: 162.8, 153.2, 152.5, 142.7, 135.9, 132.7, 129.0, 128.8, 128.3, 127.2, 125.6, 123.9, 121.3, 111.7, 110.7, 107.5, 106.9, 56.6, 55.3; HR-MS: [M − H]^+^ calculated 322.1074 *m*/*z*, found 322.1080 *m*/*z*.

*N-(3,5-Dimethoxyphenyl)-3-hydroxynaphthalene-2-carboxamide* (**2**). Yield 80%; Mp 179–184 °C; IR (cm^−1^): 3115, 1645, 1623, 1606, 1558, 1478, 1455, 1341, 1318, 1269, 1227, 1198, 1156, 1061, 948, 838, 811, 799, 767, 678; ^1^H-NMR (DMSO-*d*_6_), δ: 11.26 (s, 1H), 10.52 (s, 1H), 8.46 (s, 1H), 7.92 (d, 1H, *J* = 8.1 Hz), 7.76 (d, 1H, *J* = 8.4 Hz), 7.51 (t, 1H, *J* = 7.5 Hz), 7.36 (t, 1H, *J* = 7.3 Hz), 7.32 (s, 1H), 7.04 (d, 2H, *J* = 1.8 Hz), 6.31 (t, 1H, *J* = 1.8 Hz), 3.76 (s, 6H); ^13^C-NMR (DMSO-*d*_6_), δ: 165.7, 160.5, 153.6, 140.2, 135.7, 130.5, 128.7, 128.1, 126.9, 125.8, 123.8, 122.1, 110.5, 98.7, 96.0, 55.2; HR-MS: [M − H]^+^ calculated 322.1074 *m*/*z*, found 322.1081 *m*/*z*.

*N-(2,5-Dimethylphenyl)-3-hydroxynaphthalene-2-carboxamide* (**3**). Yield 84%; Mp 209–212 °C; IR (cm^−1^): 3328, 3013, 1633, 1621, 1597, 1577, 1538, 1519, 1493, 1446, 1413, 1361, 1326, 1287, 1262, 1221, 1174, 1067, 1000, 957, 917, 849, 800, 775, 730, 682; ^1^H-NMR (DMSO-*d*_6_), δ: 11.83 (s, 1H), 10.49 (s, 1H), 8.66 (s, 1H), 7.95 (d, 1H, *J* = 8.1 Hz), 7.80 (s, 1H), 7.78 (d, 1H. *J* = 8.4 Hz), 7.52 (t, 1H, *J* = 7.3 Hz), 7.37 (t, 1H, *J* = 7.3 Hz), 7.36 (s, 1H), 7.17 (d, 1H, *J* = 7.7 Hz), 6.94 (d, 1H, *J* = 7.7 Hz), 2.31 (s, 3H), 2.28 (s, 3H); ^13^C-NMR (DMSO-*d*_6_), δ: 164.5, 153.6, 136.2, 135.9, 135.4, 131.5, 130.2, 128.9, 128.3, 127.0, 126.9, 125.7, 125.6, 123.9, 123.9, 120.7, 110.7, 20.8, 17.4; HR-MS: [M − H]^+^ calculated 290.11756 *m*/*z*, found 290.11829 *m*/*z*.

*N-(2,6-Dimethylphenyl)-3-hydroxynaphthalene-2-carboxamide* (**4**). Yield 83%; Mp 192–196 °C; IR (cm^−1^): 3054, 1652, 1635, 1622, 1590, 1509, 1470, 1446, 1393, 1358, 1346, 1269, 1229, 1168, 1070, 953, 936, 916, 869, 842, 776, 750, 712, 671; ^1^H-NMR (DMSO-*d*_6_), δ: 11.73 (s, 1H), 10.24 (s, 1H), 8.63 (s, 1H), 7.92 (d, 1H, *J* = 7.7 Hz), 7.78 (d, 1H, *J* = 8.1 Hz), 7.53 (t, 1H, *J* = 7.3 Hz), 7.37 (t, 1H, *J* = 7.3 Hz), 7.34 (s, 1H), 7.16 (s, 3H), 2.25 (s, 6H); ^13^C-NMR (DMSO-*d*_6_), δ: 166.6, 155.0, 136.0, 135.4, 134.5, 130.0, 128.7, 128.3, 127.8, 126.9, 126.7, 125.8, 123.8, 119.7, 110.7, 18.1; HR-MS: [M − H]^+^ calculated 290.1176 *m*/*z*, found 290.1183 *m*/*z*.

*N-(3,5-Dimethylphenyl)-3-hydroxynaphthalene-2-carboxamide* (**5**). Yield 83%; Mp 184–186 °C; IR (cm^−1^): 3303, 3042, 2011, 2916, 1635, 1621, 1598, 1557, 1519, 1454, 1396, 1358, 1335, 1224, 1210, 1169, 1145, 916, 870, 838, 772, 744, 711, 685; ^1^H-NMR (DMSO-*d*_6_), δ: 11.41 (s, 1H), 10.48 (s, 1H), 8.52 (s, 1H), 7.92 (d, 1H, *J* = 8.1 Hz), 7.77 (d, 1H, *J* = 8.4 Hz), 7.52 (t, 1H, *J* = 7.1 Hz), 7.40 (s, 2H), 7.36 (t, 1H, *J* = 7.3 Hz), 7.33 (s, 1H), 6.79 (s, 1H), 2.29 (s, 6H); ^13^C-NMR (DMSO-*d*_6_), δ: 165.6, 153.9, 138.2, 137.8, 135.8, 130.5, 128.7, 128.2, 126.9, 125.8, 125.6, 123.8, 121.5, 118.3, 110.6, 21.1; HR-MS: [M − H]^+^ calculated 290.1176 *m*/*z*, found 290.1185 *m*/*z*.

*N-(2,5-Difluorophenyl)-3-hydroxynaphthalene-2-carboxamide* (**6**). Yield 53%; Mp 264 °C; IR (cm^−1^): 3057, 1652, 1630, 1598, 1561, 1516, 1489, 1444, 1391, 1360, 1346, 1285, 1272, 1245, 1222, 1184, 1145, 1064, 1015, 972, 949, 908, 889, 862, 833, 807, 795, 765, 736, 716; ^1^H-NMR (DMSO-*d*_6_) δ: 11.93 (s, 1H); 11.11 (s, 1H), 8.68 (s, 1H), 8.30 (ddd, 1H, *J* = 3.2 Hz, *J* = 6.2 Hz, *J* = 10.3 Hz), 7.99 (d, 1H, *J* = 8.2 Hz), 7.78 (d, 1H, *J* = 8.3 Hz), 7.54 (ddd, 1H, *J* = 1.1 Hz, *J* = 6.8 Hz, *J* = 8.3 Hz), 7.41 (ddd, 1H, *J* = 5.1 Hz, *J* = 9.1 Hz, *J* = 10.6 Hz), 7.38 (ddd, 1H, *J* = 1.2 Hz, *J* = 6.8 Hz, *J* = 8.2 Hz), 7.37 (s, 1H), 7.00–7.04 (m, 1H); ^13^C-NMR (DMSO-*d*_6_), δ: 163.6, 157.9 (dd, *J* = 1.7 Hz, *J* = 238.4 Hz), 152.5, 148.8 (dd, *J* = 2.4 Hz, *J* = 239.1 Hz), 136.1, 132.7, 129.1, 128.6, 127.6 (t, *J* = 12.4 Hz), 127.2, 125.7, 124.1, 120.4, 116.1 (dd, *J* = 10.0 Hz, *J* = 22.1 Hz), 110.9, 110.4 (dd, *J* = 8.0 Hz, *J* = 24.3 Hz), 108.7 (d, *J* = 29.9 Hz); HR-MS: [M + H]^+^ calculated 300.0830 *m*/*z*, found 300.0832 *m*/*z.*

*N-(2,6-Difluorophenyl)-3-hydroxynaphthalene-2-carboxamide* (**7**). Yield 78%; Mp 194–198 °C; IR (cm^−1^): 3357, 1662, 1628, 1596, 1511, 1466, 1304, 1285, 1213, 1147, 1134, 1009, 899, 830, 788, 778, 747; ^1^H-NMR (DMSO-*d*_6_), δ: 11.48 (s, 1H), 10.41 (s, 1H), 8.61 (s, 1H), 7.94 (d, 1H, *J* = 8.1 Hz), 7.78 (d, 1H, *J* = 8.1 Hz), 7.54 (t, 1H, *J* = 7.0 Hz), 7.33–7.44 (m, 4H), 7.25 (t, 1H, *J* = 7.9 Hz); ^13^C-NMR (DMSO-*d*_6_), δ: 166.0, 157.9 (dd, *J* = 247.3 Hz, *J* = 5.3 Hz), 154.1, 136.2, 131.2, 128.9, 128.7 (t, *J* = 19.8 Hz), 128.5, 126.8, 125.9, 125.8, 123.9, 119.4, 114.2 (t, *J* = 16.7 Hz), 112.0 (m); HR-MS: [M − H]^+^ calculated 298.0674 *m*/*z*, found 298.0681 *m*/*z*.

*N-(3,5-Difluorophenyl)-3-hydroxynaphthalene-2-carboxamide* (**8**). Yield 79%; Mp 273–276 °C; IR (cm^−1^): 3103, 1645, 1626, 1608, 1574, 1564, 1479, 1439, 1345, 1308, 1268, 1225, 1208, 1169, 1118, 999, 989, 855, 829, 767, 740; ^1^H-NMR (DMSO-*d*_6_), δ: 11.05 (s, 1H), 10.80 (s, 1H), 8.38 (s, 1H), 7.93 (d, 1H, *J* = 8.0 Hz), 7.76 (d, 1H, *J* = 8.4 Hz), 7.51–7.62 (m, 3H), 7.38 (t, 1H, *J* = 7.5 Hz), 7.34 (s, 1H), 6.99 (t, 1H, *J* = 8.0 Hz); ^13^C-NMR (DMSO-*d*_6_), δ: 165.8, 162.4 (dd, *J* = 241.3 Hz, *J* = 15.2 Hz), 153.0, 141.2 (t, *J* = 13.7 Hz), 135.7, 130.6, 128.7, 128.2, 126.9, 125.8, 123.8, 122.8, 110.5, 103.0 (m), 98.93 (t, *J* = 25.8 Hz); HR-MS: [M − H]^+^ calculated 298.0674 *m*/*z*, found 298.0685 *m*/*z*.

*N-(2,5-Dichlorophenyl)-3-hydroxynaphthalene-2-carboxamide* (**9**). Yield 72%; Mp 252 °C; IR (cm^−1^): 3187, 1637, 1623, 1590, 1580, 1519, 1470, 1455, 1404, 1359, 1347, 1313, 1265, 1204, 1174, 1140, 1090, 1073, 1047, 1017, 979, 960, 916, 867, 846, 808, 769, 750, 707; ^1^H-NMR (DMSO-*d*_6_) δ: 12.03 (s, 1H), 11.28 (s, 1H), 8.71 (s, 1H), 8.67 (d, 1H, *J* = 2.5 Hz), 7.99 (d, 1H, *J* = 8.2 Hz), 7.78 (d, 1H, *J* = 8.2 Hz), 7.60 (d, 1H, *J* = 8.6 Hz), 7.53 (ddd, 1H, *J* = 1.2 Hz, *J* = 6.8 Hz, *J* = 8.3 Hz), 7.38 (s, 1H), 7.37 (ddd, 1H, *J* = 1.1 Hz, *J* = 6.8 Hz, *J =* 8.2 Hz), 7.25 (dd, 1H, *J* = 2.6 Hz, *J* = 8.6 Hz); ^13^C-NMR (DMSO-*d*_6_), δ: 163.5, 152.4, 136.5, 136.1, 133.0, 132.1, 130.6, 129.1, 128.7, 127.2, 125.7, 124.5, 124.0, 121.39, 121.35, 120.3, 110.9; HR-MS: [M + H]^+^ calculated 332.0240 *m*/*z*, found 332.0243 *m*/*z.*

*N-(2,6-Dichlorophenyl)-3-hydroxynaphthalene-2-carboxamide* (**10**). Yield 73%; Mp 283–284 °C; IR (cm^−1^): 3209, 1623, 1614, 1570, 1515, 1463, 1450, 1433, 1405, 1328, 1236, 1205, 1155, 970, 912, 818, 793, 783, 771, 745, 704, 679; ^1^H-NMR (DMSO-*d*_6_) δ: 11.57 (s, 1H), 10.66 (s, 1H), 8.66 (s, 1H), 7.95 (d, 1H, *J* = 8.2 Hz), 7.79 (d, 1H, *J* = 8.2 Hz,), 7.62 (d, 2H, *J* = 8.2 Hz), 7.53 (ddd, 1H, *J* = 7.8 Hz, *J* = 6.9 Hz, 1.0 Hz), 7.43 (t, 1H, *J* = 8.2 Hz), 7.37 (ddd, 1H, *J* = 8.2 Hz, *J* = 6.9 Hz, *J* = 0.9 Hz), 7.35 (s, 1H); ^13^C-NMR (DMSO-*d*_6_), δ: 165.8, 153.6, 136.0, 134.1, 133.3, 131.1, 129.3, 128.9, 128.8, 128.2, 126.8, 125.9, 125.6, 123.9, 120.4; HR-MS: [M + H]^+^ calculated 332.0240 *m*/*z*, found 332.0238 *m*/*z*.

*N-(3,4-Dichlorophenyl)-3-hydroxynaphthalene-2-carboxamide* (**11**). Yield 76%; Mp 278–279 °C; IR (cm^−1^): 3080, 1622, 1605, 1589, 1566, 1545, 1474, 1449, 1396, 1377, 1358, 1344, 1240, 1207, 1172, 1134, 1073, 1024, 955, 925, 898, 852, 837, 826, 771, 748, 680; ^1^H-NMR (DMSO-*d*_6_) δ: 11.09 (s, 1H), 10.75 (s, 1H), 8.41 (s, 1H), 8.18 (d, 1H, *J* = 2.3 Hz), 7.93 (d, 1H, *J* = 8.2 Hz), 7.77 (d, 1H, *J* = 8.2 Hz), 7.72 (dd, 1H, *J* = 8.9 Hz, *J* = 12.3 Hz), 7.64 (d, 1H, *J* = 8.7 Hz), 7.51 (ddd, 1H, *J* = 7.8 Hz, *J* = 6.9 Hz, *J* = 0.9 Hz), 7.36 (ddd, 1H, *J* = 8.2 Hz, *J* = 6.9 Hz, *J* = 0.9 Hz), 7.33 (s, 1H); ^13^C-NMR (DMSO-*d*_6_), δ: 165.8, 153.2, 138.8, 135.7, 131.0, 130.7, 130.5,128.7, 128.2, 126.9, 125.8, 125.3, 123.8, 122.5, 121.4, 120.3, 110.5; HR-MS: [M + H]^+^ calculated 332.0240 *m*/*z*, found 332.0242 *m*/*z*.

*N-(3,5-Dichlorophenyl)-3-hydroxynaphthalene-2-carboxamide* (**12**). Yield 70%; Mp 252 °C; IR (cm^−1^): 3087, 1646, 1630, 1583, 1547, 1447, 1409, 1396, 1375, 1345, 1329, 1269, 1254, 1204, 1172, 1147, 1115, 1092, 1072, 1017, 993, 938, 917, 905, 867, 861, 836, 802, 788, 764, 741, 723, 688; ^1^H-NMR (DMSO-*d*_6_) δ: 11.04 (s, 1H), 10.75 (s, 1H), 8.39 (s, 1H), 7.92 (d, 1H, *J* = 8.2 Hz), 7.89 (d, 2H, *J* = 1.7 Hz), 7.77 (d, 1H, *J* = 8.3 Hz), 7.51 (ddd, 1H, *J* = 1.2 Hz, *J* = 6.8 Hz, *J* = 8.3 Hz), 7.36 (ddd, 1H, *J* = 1.1 Hz, *J* = 6.8 Hz, *J* = 8.2 Hz), 7.34 (t, 1H, *J* = 1.7 Hz), 7.34 (s, 1H); ^13^C-NMR (DMSO-*d*_6_), δ: 165.9, 153.1, 141.0, 135.7, 134.1, 130.5, 128.7, 128.2, 126.8, 125.8, 123.8, 123.0, 122.6, 118.3, 110.5; HR-MS: [M + H]^+^ calculated 332.0240 *m*/*z*, found 332.0244 *m*/*z*.

*N-[3,5-bis(Trifluoromethyl)phenyl]-3-hydroxynaphthalene-2-carboxamide* (**13**). Yield 57%; Mp 232–235 °C; IR (cm^−1^): 3256, 1651, 1631, 1601, 1578, 1568, 1473, 1384, 1357, 1346, 1274, 1162, 1120, 1113, 943, 883, 866, 836, 765, 744, 707, 682; ^1^H-NMR (DMSO-*d*_6_), δ: 11.05 (s, 1H), 10.99 (s, 1H), 8.50 (s, 2H), 8.41 (s, 1H), 7.93 (d, 1H, *J* = 8.1 Hz), 7.84 (s, 1H), 7.78 (d, 1H, *J* = 8.4 Hz), 7.52 (t, 1H, *J* = 6.6 Hz), 7.34–7.40 (m, 2H); ^13^C-NMR (DMSO-*d*_6_), δ: 166.4, 153.2, 140.6, 135.8, 130.8 (q, *J* = 32.8 Hz), 130.6, 128.6, 128.2, 126.8, 125.9, 123.9, 123.3 (q, *J* = 272.4 Hz), 122.6, 120.0 (q, *J* = 3.6 Hz), 116.6 (sep, *J* = 3.4 Hz), 110.5; HR-MS: [M − H]^+^ calculated 398.0610 *m*/*z*, found 398.0620 *m*/*z*.

*3-Hydroxy-N-[4-methyl-3-(trifluoromethyl)phenyl]naphthalene-2-carboxamide* (**14**). Yield 68%; Mp 251–252 °C; IR (cm^−1^): 3135, 1638, 1607, 1553, 1502, 1450, 1398, 1361, 1316, 1204, 1169, 1141, 1113, 1052, 1008, 955, 920, 900, 867, 837, 770, 749, 726; ^1^H-NMR (DMSO-*d*_6_) δ: 11.19 (s, 1H), 10.73 (s, 1H), 8.46 (s, 1H), 8.20 (d, 1H, *J* = 1.9 Hz), 7.92 (d, 1H, *J* = 8.2 Hz), 7.89 (dd, 1H, *J* = 1.9 Hz, *J* = 8.3 Hz), 7.77 (d, 1H, *J* = 8.3 Hz), 7.51 (ddd, 1H, *J* = 1.2 Hz, *J* = 6.8 Hz, *J* = 8.3 Hz), 7.44 (d, 1H, *J* = 8.3 Hz), 7.36 (ddd, 1H, *J* = 1.1 Hz, *J* = 6.8 Hz, *J* = 8.2 Hz), 7.33 (s, 1H), 2.42 (s, 3H); ^13^C-NMR (DMSO-*d*_6_), δ: 166.0, 153.6, 136.8, 135.8, 132.7, 131.1 (q, *J* = 1.7 Hz), 130.4, 128.7, 128.2, 127.5 (q, *J* = 29.3 Hz), 126.9, 125.8, 124.4 (q, *J* = 273.8 Hz), 123.8, 122.0, 117.4 (q, *J* = 5.9 Hz), 110.6, 18.3 (q, *J* = 1.9 Hz); HR-MS: [M + H]^+^ calculated 346.1049 *m*/*z*, found 346.1055 *m*/z.

*N-[4-Fluoro-3-(trifluoromethyl)phenyl]-3-hydroxynaphthalene-2-carboxamide* (**15**). Yield 72%; Mp 231–232 °C; IR (cm^−1^): 3101, 1639, 1613, 1575, 1498, 1453, 1421, 1397, 1361, 1344, 1321, 1273, 1254, 1229, 1206, 1179, 1144, 1123, 1053, 959, 925, 903, 868, 839, 814, 771, 753, 732, 679; ^1^H-NMR (DMSO-*d*_6_) δ: 11.10 (s, 1H), 10.81 (s, 1H), 8.43 (s, 1H), 8.29 (dd, 1H, *J* = 2.6 Hz, *J* = 6.5 Hz), 8.05 (ddd, 1H, *J* = 3.0 Hz, *J* = 4.2 Hz, *J* = 8.9 Hz), 7.92 (d, 1H, *J* = 8.2 Hz), 7.77 (d, 1H, *J* = 8.3 Hz), 7.55 (dd, 1H, *J* = 9.0 Hz, *J* = 10.5 Hz), 7.51 (ddd, 1H, *J* = 1.2 Hz, *J* = 6.8 Hz, 8.3 Hz), 7.36 (ddd, 1H, *J* = 1.2 Hz, *J* = 6.8 Hz, *J* = 8.2 Hz), 7.34 (s, 1H); ^13^C-NMR (DMSO-*d*_6_), δ: 166.0, 154.8 (dq, *J* = 2.1 Hz, *J* = 250.3 Hz), 153.4, 135.8, 135.4 (d, *J*= 2.9 Hz), 130.4, 128.7, 128.2, 126.8, (d, *J*= 8.1 Hz), 126.5, 125.8, 123.8, 122.5 (dq, *J* = 1.1, 272.1 Hz), 122.2, 118.4 (q, *J* = 4.9 Hz), 117.7 (d, *J* = 21.4 Hz), 116.5 (dq, *J* = 13.4, 32.4 Hz), 110.5; HR-MS: [M + H]^+^ calculated 350.0799 *m*/*z*, found 350.0801 *m*/z.

*N-[4-Bromo-3-(trifluoromethyl)phenyl]-3-hydroxynaphthalene-2-carboxamide* (**16**). Yield 62%; Mp 234–235 °C; IR (cm^−1^): 3161, 1637, 1622, 1602, 1549, 1478, 1451, 1395, 1359, 1313, 1264, 1208, 1176, 1151, 1127, 1105, 1021, 957, 924, 896, 867, 836, 770, 750, 721; ^1^H-NMR (DMSO-*d*_6_) δ: 11.07 (s, 1H), 10.86 (s, 1H), 8.42 (s, 1H), 8.37 (d, 1H, *J* = 2.5 Hz), 7.97 (dd, 1H, *J* = 2.5 Hz, *J* = 8.7 Hz), 7.92 (d, 1H, *J* = 8.2 Hz), 7.88 (d, 1H, *J* = 8.7 Hz), 7.77 (d, 1H, *J* = 8.3 Hz), 7.51 (ddd, 1H, *J* = 1.2 Hz, *J* = 6.8 Hz, *J* = 8.3 Hz), 7.36 (ddd, 1H, *J* = 1.2 Hz, *J* = 6.8 Hz, *J* = 8.2 Hz), 7.34 (s, 1H); ^13^C-NMR (DMSO-*d*_6_), δ: 166.1, 153.3, 138.6, 135.8, 135.5, 130.5, 128.7, 128.6 (q, *J* = 30.5 Hz), 128.2, 126.9, 125.8, 125.1, 123.8, 122.9 (q, *J =* 273.1 Hz), 122.5, 119.3 (q, *J* = 5.8 Hz), 112.2 (q, *J* = 1.8 Hz), 110.5; HR-MS: [M + H]^+^ calculated 409.9998 *m*/*z*, found 410.0005 *m*/z.

*N-(4-Bromo-3-fluorophenyl)-3-hydroxynaphthalene-2-carboxamide* (**17**). Yield 72%; Mp 255–256 °C; IR (cm^−1^): 3053, 1634, 1619, 1599, 1557, 1486, 1447, 1400, 1356, 1344, 1270, 1244, 1221, 1207, 1168, 1145, 1128, 1078, 1043, 963, 915, 871, 844, 817, 771, 749, 704; ^1^H-NMR (DMSO-*d*_6_) δ: 11.10 (s, 1H), 10.78 (s, 1H), 8.41 (s, 1H), 7.95 (dd, 1H, *J* = 2.3 Hz, *J* = 11.4 Hz), 7.93 (d, 1H, *J* = 8.3 Hz), 7.76 (d, 1H, *J* = 8.3 Hz), 7.69 (dd, 1H, *J* = 8.2 Hz, *J* = 8.7 Hz), 7.52 (ddd, 1H, *J* = 0.7 Hz, *J* = 2.4 Hz, *J* = 8.9 Hz), 7.51 (ddd, 1H, *J* = 1.3 Hz, *J* = 6.8 Hz, *J* = 8.3 Hz), 7.36 (ddd, 1H, *J* = 1.2 Hz, *J* = 6.8 Hz, *J* = 8.2 Hz), 7.33 (s, 1H); ^13^C-NMR (DMSO-*d*_6_), δ: 165.8, 158.1 (d, *J* = 242.4 Hz), 153.2, 139.9 (d, *J* = 10.2 Hz), 135.7, 133.3 (d, *J* = 1.2 Hz), 130.6, 128.7, 128.2, 126.9, 125.8, 123.8, 122.5, 101.5 (d, *J* = 21.0 Hz), 108.2 (d, *J* = 27.1 Hz), 117.6 (d, *J* = 3.0 Hz), 110.5; HR-MS: [M + H]^+^ calculated 360.0030 *m*/*z*, found 360.0036 *m*/z.

*N-[2-Fluoro-5-(trifluoromethyl)phenyl]-3-hydroxynaphthalene-2-carboxamide* (**18**). Yield 77%; Mp 222–223 °C; IR (cm^−1^): 3276, 1656, 1632, 1619, 1568, 1521, 1498, 1468, 1442, 1396, 1345, 1321, 1263, 1248, 1215, 1204, 1165, 1148, 1126, 1072, 926, 917, 904, 888, 865, 812, 765, 746, 716; ^1^H-NMR (DMSO-*d*_6_) δ: 11.92 (s, 1H), 11.16 (s, 1H), 8.83 (dd, 1H, *J* = 1.6 Hz, *J* = 7.1 Hz), 8.67 (s, 1H), 7.98 (d, 1H, *J* = 8.2 Hz), 7.57–7.62 (m, 2H), 7.78 (d, 1H, *J* = 8.3 Hz), 7.53 (ddd, 1H, *J* = 1.2 Hz, *J* = 6.8 Hz, *J* = 8.3 Hz), 7.38 (ddd, 1H, *J* = 1.1 Hz, *J* = 6.8 Hz, *J* = 8.2 Hz), 7.38 (s, 1H); ^13^C-NMR (DMSO-*d*_6_), δ: 164.0, 154.5 (d, *J* = 249.9 Hz), 152.6, 136.1, 132.7, 129.1, 128.7, 127.5 (d, *J* = 11.5 Hz), 125.8, 127.2, 125.5 (dq, *J* = 3.1 Hz, *J* = 32.2 Hz), 124.1, 123.8 (q, *J* = 272.3 Hz), 121.9 (dq, *J* = 3.9 Hz, 8.7 Hz), 120.4, 118.9 (m), 116.4 (d, *J* = 20.9 Hz), 110.9; HR-MS: [M + H]^+^ calculated 350.0799 *m*/*z*, found 350.0802 *m*/z.

*N-[3-Fluoro-5-(trifluoromethyl)phenyl]-3-hydroxynaphthalene-2-carboxamide* (**19**). Yield 66%; Mp 245–247 °C; IR (cm^−1^): 3094, 1647, 1623, 1575, 1564, 1482, 1458, 1436, 1397, 1358, 1347, 1327, 1254, 1217, 1207, 1171, 1149, 1128, 1099, 1003, 995, 922, 894, 875, 867, 858, 840, 799, 766, 745; ^1^H-NMR (DMSO-*d*_6_) δ: 11.01 (s, 1H); 10.93 (s, 1H); 8.40 (s, 1H); 8.05 (s, 1H); 8.00 (d, 1H, *J* = 11.0 Hz); 7.93 (d, 1H, *J* = 8.2 Hz); 7.77 (d, 1H, *J* = 8.2 Hz); 7.51 (dd, 1H, *J* = 8.2 Hz, 6.9 Hz); 7.43 (d, 1H, *J* = 8.2 Hz); 7.37 (t, 1H, *J* = 7.2 Hz); 7.34 (s, 1H); ^13^C-NMR (DMSO-*d*_6_), δ: 166.1; 162.1 (d, *J* = 244.2 Hz); 153.1; 141.4 (d, *J* = 11.6 Hz); 135.8; 131.0 (td, *J* = 33.2 Hz, 10.1 Hz); 130.6; 128.7; 128.2; 126.9; 125.9; 123.8; 123.3 (qd, *J* = 273.1 Hz, 4.3 Hz); 122.8; 112.6 (m); 110.5 (d, *J* = 26.0 Hz); 110.5; 107.5 (m); HR-MS: [M + H]^+^ calculated 350.0799 *m*/*z*, found 350.0791 *m*/z.

*N-[2-Chloro-5-(trifluoromethyl)phenyl]-3-hydroxynaphthalene-2-carboxamide* (**20**). Yield 60%; Mp 185–188 °C; IR (cm^−1^): 3193, 1642, 1622, 1601, 1586, 1544, 1519, 1425, 1329, 1253, 1204, 1167, 1150, 1119, 1080, 1046, 916, 895, 873, 848, 826, 770, 752; ^1^H-NMR (DMSO-*d*_6_) δ: 12.09 (s, 1H), 11.41 (s, 1H), 8.98 (d, 1H, *J* = 2.2 Hz), 8.72 (s, 1H), 7.99 (d, 1H, *J* = 8.1 Hz), 7.82 (d, 1H, *J* = 7.7 Hz), 7.78 (d, 1H, *J* = 8.1 Hz), 7.50–7.57 (m, 2H), 7.33–7.41 (m, 2H); ^13^C-NMR (DMSO-*d*_6_), δ: 163.7, 152.5, 136.3, 136.2, 133.1, 130.5, 129.2, 128.7, 128.4 (q, *J* = 32.0 Hz), 127.1, 126.8 (q, *J* = 1.5 Hz), 125.7, 124.1, 123.7 (q, *J* = 272.4 Hz), 121.3 (q, *J* = 3.9 Hz), 120.3, 118.2 (q, *J* = 3.8 Hz), 110.9; HR-MS: [M − H]^+^ calculated 364.0347 *m*/*z*, found 364.0355 *m*/*z*.

*N-[3-Fluoro-4-(trifluoromethyl)phenyl]-3-hydroxynaphthalene-2-carboxamide* (**21**). Yield 61%; Mp 273–274 °C; IR (cm^−1^): 3061, 1622, 1605, 1557, 1451, 1432, 1417, 1398, 1360, 1342, 1317, 1271, 1244, 1210, 1174, 1120, 1069, 1047, 977, 962, 945, 915, 873, 860, 842, 815, 772, 754, 732, 702; ^1^H NMR (CDCl_3_) δ: 11.05 (s, 1H), 10.96 (s, 1H), 8.39 (s, 1H), 8.02 (dd, 1H, *J* = 1.4 Hz, *J* = 13.5 Hz), 7.94 (d, 1H, *J* = 8.2 Hz), 7.78 (t, 1H, *J* = 8.6 Hz), 7.77 (d, 1H, *J* = 8.3 Hz), 7.71 (dd, 1H, *J* = 1.4 Hz, *J* = 8.7 Hz), 7.51 (ddd, 1H, *J* = 1.2 Hz, *J* = 6.8 Hz, *J* = 8.3 Hz), 7.36 (ddd, 1H, *J* = 1.2, *J* = 6.8 Hz, *J* = 8.2 Hz), 7.34 (s, 1H); ^13^C-NMR (DMSO-*d*_6_), δ: 166.0, 159.2 (dq, *J* = 2.0 Hz, *J* = 250.6 Hz), 152.9, 144.5 (d, *J* = 11.4 Hz), 135.8, 130.7, 107.5 (d, *J* = 25.4 Hz), 128.7, 128.2, 127.8 (m), 126.9, 125.8, 123.8, 122.9, 122.8 (q *J* = 271.1 Hz), 115.6 (d, *J* = 1.4 Hz, *J* = 8.7 Hz), 111.0 (dq, *J* = 12.5, 32.6 Hz), 110.5; HR-MS: [M + H]^+^ calculated 350.0799 *m*/*z*, found 350.0801 *m*/*z*.

*N-[2-Bromo-4-(trifluoromethyl)phenyl]-3-hydroxynaphthalene-2-carboxamide* (**22**). Yield 67%; Mp 231–233 °C; IR (cm^−1^): 3225, 3093, 1643, 1626, 1601, 1588, 1541, 1492, 1451, 1404, 1359, 1319, 1274, 1212, 1169, 1120, 1078, 1042, 955, 919, 894, 871, 838, 803;^1^H NMR (CDCl_3_) δ: 12.09 (s, 1H); 11.32 (s, 1H); 8.76 (d, 1H, *J* = 8.9 Hz); 8.73 (s, 1H); 8,12 (s, 1H); 8.01 (d, 1H, *J* = 8.2 Hz); 7.85 (dd, 1H, *J* = 8.2 Hz, *J* = 1.4 Hz); 7.79 (d, 1H, *J* = 8.2 Hz); 7.56–7.53 (m, 1H); 7.40 (s, 1H); 7.39–7.73 (m, 1H); ^13^C-NMR (DMSO-*d*_6_), δ: 163.6; 152.5; 140.4; 136.2; 133.2; 129.6 (q, *J* = 4.3 Hz); 129.2; 128.8; 127.2; 125.7; 125.7 (q, *J* = 4.3 Hz); 125.2 (q, *J* = 33.2 Hz); 124.1; 123.4 (q, *J* = 271.7 Hz); 122.4; 120.3; 113.4; 110.9; HR-MS: [M + H]^+^ calculated 409.9998 *m*/*z*, found 409.9988 *m*/*z*.

### 3.3. Biological Testing

#### 3.3.1. In Vitro Antibacterial Evaluation

The synthesized compounds were evaluated for in vitro antibacterial activity against representatives of multidrug-resistant bacteria and clinical isolates of methicillin-resistant *Staphylococcus aureus* (MRSA) 63718, SA 630, and SA 3202 that were obtained from the National Institute of Public Health (Prague, Czech Republic) [41]. *S. aureus* ATCC 29213 was used as a reference and quality control strain. Ampicillin and ciprofloxacin (Sigma, St. Louis, MO, USA) were employed as the standard. Prior to testing, each strain was passaged onto nutrient agar (Oxoid, Basingstoke, UK) with 5% of bovine blood, and bacterial inocula were prepared by suspending a small portion of a bacterial colony in sterile phosphate-buffered saline (pH 7.2–7.3). The cell density was adjusted to 0.5 McFarland units using a densitometer (Densi-La-Meter, LIAP, Riga, Latvia). This inoculum was diluted to reach the final concentration of bacterial cells 5 × 10^5^ CFU/mL in the wells. The compounds were dissolved in DMSO (Sigma), and the final concentration of DMSO in the cation-adjusted Mueller–Hinton (CaMH) broth (Oxoid) did not exceed 2.5% of the total solution composition. The final concentrations of the evaluated compounds ranged from 256 to 0.008 μg/mL. The broth dilution micro-method, modified according to the NCCLS (National Committee for Clinical Laboratory Standards) guidelines in Mueller–Hinton (MH) broth, was used to determine the minimum inhibitory concentration (MIC) [42]. Drug-free controls, sterility controls, and controls consisting of MH broth and DMSO alone were included. The determination of results was performed visually after 24 h of static incubation in the darkness at 37 °C in an aerobic atmosphere. The results are reported in Table 1.

#### 3.3.2. In Vitro Antimycobacterial Assessment

*Mycobacterium tuberculosis* ATCC 25177/H37Ra was grown in Middlebrook broth (MB), supplemented with Oleic-Albumin-Dextrose-Catalase (OADC) supplement (Difco, Lawrence, KS, USA). At log phase growth, a culture sample (10 mL) was centrifuged at 15,000 rpm/20 min using a bench top centrifuge (MPW-65R, MPW Med Instruments, Warszawa, Poland). Following the removal of the supernatant, the pellet was washed in fresh Middlebrook 7H9GC broth and resuspended in fresh, ODAC-supplemented MB (10 mL). The turbidity was adjusted to match McFarland standard No. 1 (3 × 10^8^ CFU) with MB broth. A further 1:10 dilution of the culture was then performed in MB broth. The antimicrobial susceptibility of *M. tuberculosis* was investigated in a 96-well plate format. In these experiments, sterile deionized water (300 µL) was added to all outer-perimeter wells of the plates to minimize evaporation of the medium in the test wells during incubation. Each evaluated compound (100 µL) was incubated with *M. tuberculosis* (100 µL). Dilutions of each compound were prepared in duplicate. For all synthesized compounds, final concentrations ranged from 128 to 4 μg/mL. All compounds were dissolved in DMSO, and subsequent dilutions were made in supplemented MB. The plates were sealed with Parafilm and incubated at 37 °C for 14 days. Following incubation, a 10% addition of alamarBlue (Difco) was mixed into each well, and readings at 570 nm and 600 nm were taken, initially for background subtraction and subsequently after 24 h reincubation. The background subtraction is necessary for strongly colored compounds, where the color may interfere with the interpretation of any color change. For noninterfering compounds, a blue color in the well was interpreted as the absence of growth, and a pink color was scored as growth. Isoniazid and rifampicin (Sigma) used as positive control, as it is a clinically used antitubercular drug. The results are shown in Table 1.

#### 3.3.3. MTT Assay

The percent inhibition was determined through the MTT assay. Compounds were prepared according to the previously described procedure and diluted in MB broth for *M. tuberculosis* ATCC 25177/H37Ra. *M. tuberculosis* ATCC 25177/H37Ra was suspended in ODAC supplemented MB at 1.0 McFarland and then diluted 1:10, using MB as a diluent. The diluted mycobacteria (50 µL) were added to each well containing the compound to be investigated. Diluted mycobacteria in broth free from inhibiting compounds were employed as the growth control. All compounds were prepared in duplicate. Plates were incubated at 37 °C for 7 days for *M. tuberculosis*. After the incubation period, 10% well volume of MTT (3-(4,5-dimethylthiazol-2-yl)-2,5-diphenyltetrazolium bromide) reagent (Sigma) was added into each well and the plates were incubated at 37 °C for 4 h in dark for mycobacteria. Subsequently, 100 µL of 17% sodium dodecyl sulfate in 40% dimethylformamide was added to each well. The plates were read at 570 nm. The absorbance readings for cells grown in the presence of the tested compounds were compared with those representing uninhibited cell growth to specify the relative percent inhibition. The percent viability is calculated through the comparison of a measured value and that of the uninhibited control: % viability = OD_570E_/OD_570P_ × 100, where OD_570E_ is the reading from the compound-exposed cells, while OD_570P_ is the reading from the uninhibited cells (positive control). Cytotoxic potential is evaluated by percent viability <70% [44,45,46,64].

#### 3.3.4. Combined Effect with Clinically Used Drugs

For the synergy effect study, a method of fractional inhibitory concentration was engaged. A tested compound (A) and antibiotic (B) (ciprofloxacin and oxacillin, purchased from Sigma) were diluted in the microtitration plate in cation adjusted Mueller–Hinton (CaMH) broth to get an original combination of concentrations in every well. Raw H was employed for the evaluation of MIC(A); column 12 was applied for assessment of MIC(B). The plate was inoculated by the bacterial suspension to reach a final concentration of 5 × 10^5^ CFU/mL in the wells. The fractional inhibitory concentration index (FICI) was measured using the concentrations in the first nonturbid (clear) well found in each row and column along with the turbidity/nonturbidity interface. To interpret the combined effect, the lowest FICI was used [60]. A ΣFICI ≤ 0.5 means synergy; 0.5 < ΣFICI < 1 means additivity; 1 ≤ ΣFICI < 4 means indifference; and ΣFICI ≥ 4 means antagonism, respectively [61]. The tests were made in duplicate, and the results were averaged. The results are reported in Table 2.

#### 3.3.5. Dynamic of Antibacterial Activity

The method of time-kill curves was employed to investigate the bactericidal effect of the specified compounds [65]. The experiment was conducted with *S. aureus* ATCC 29213 and MRSA SA 3202 isolate. The compounds were diluted in CaMH broth to reach concentrations equal to 1×MIC, 2×MIC, and 4×MIC, respectively. The tubes were inoculated by bacterial inoculum in the exponential phase of growth to get a final concentration of 7.5 × 10^6^ CFU/mL. The tubes were incubated statically at 37 °C. Immediately after inoculation and after 4, 6, 8, and 24 h, 100 µL of the sample was serially diluted (1:10) in phosphate-buffered saline. Subsequently, 2 × 20 µL from each dilution were put onto an MH agar plate and cultivated at 37 °C for 24 h. After incubation, the CFUs of dilutions containing 5–50 colonies were counted. Results were expressed as a decrease of log_10_(CFU) in each time compared to the starting inoculum. Bactericidal effect is specified as a −3log decrease of CFU/mL compared to the growth control at timepoint 0. The test was performed in duplicate on two separate occasions, and the results were averaged. The growth curves with error bars are illustrated in Figure 6.

### 3.4. Theoretical Calculations

#### 3.4.1. Model Building and Molecular Modeling

CACTVS/csed and CORINA editors were engaged to produce each structural model and its initial spatial geometry. OpenBabel (inter)change file format converter was employed for data conversion as well. Sybyl-X 2.0/Certara software package running on an HP Z200 workstation with a Debian 10.0 operating system was used to conduct the molecular modeling simulations. The initial compound geometry optimization with MAXMIN2 module was performed using the standard Tripos force field (POWELL conjugate gradient algorithm) with a 0.01 kcal/mol energy gradient convergence criterion. The specification of the electrostatic potential values based on the partial atomic charges was carried out with the Gasteiger–Hückel method implemented in Sybyl-X. One 13-ordered atom trial alignment on the most active molecule **13** according to the active analog approach (AAA) was used in the FIT method to cover the entire bonding topology in the maximal common structure (MCS).

SONNIA software was applied for simulation of 10 × 10 to 30 × 30 SOMs with a winning distance ranging from 0.2 to 2.0 in CoMSA analysis. Cartesian coordinates of the molecular surfaces were used as input to Kohonen SOM network to generate a 2D map of the electrostatic potential (MEP) for the set of superimposed molecules. The output maps were reshaped into a 100- to 900-element vectors that were subsequently transformed by the PLS method implemented in the MATLAB environment [66].

#### 3.4.2. Similarity-Driven Activity Landscape

The quantitative sampling of similarity-related activity landscape delivers a subtle picture of favorable and disallowed structural modifications that are valid for the specification of the activity cliffs [11]. The smoothness of this surface can be numerically quantified using structure-activity landscape index (SALI) calculated according to the following formula:

(1) $$SALI_{x,y}=\frac{\left|A_{x}-A_{y}\right|}{1-sim(x,y)}$$

where $A_{x}$ and $A_{y}$ are activity profiles for the *x*-th and *y*-th molecule and *sim*(*x,y*) is the similarity estimation between the corresponding molecules [13,14,15,16]. The combination of the pairwise dissimilarity and absolute discrepancies in the molecular activities assigns the score to the specific spot of the response surface. The negligible impact of the specific similarity metric on the overall appearance of the symmetric SALI matrix has been observed; therefore, Tanimoto coefficient was applied in the fingerprint-based similarity analysis [14]. In this case, the structural relatedness between molecules library is usually estimated using a function mapping (dis)similarities between the pairs of bitstring descriptors given by the following equation:

(2) $$T(x,y)=\frac{n_{xy}}{\left(n_{x}+n_{y}-n_{xy}\right)}$$

where $n_{xy}$ is the number of bits set into 1 shared in the fingerprint of the molecule *x* and *y*, $n_{x}$ is the number of bits set into 1 in the molecule *x*, $n_{y}$ is the number of bits set into 1 in the molecule *y*, respectively. Top-ranked objects are supposed to have similar properties, although the validity of this assumption is fairly questionable because 70% of compounds with *T*(*x*,*y*) > 0.85 to an active analog have a comparatively low opportunity of being active in the same way [67]. A relatively sparse sampling of chemistry space may be sufficient to identify the potential activity cliffs.

#### 3.4.3. Selection-Driven Surface Analysis

The hypothesis-based similarity in a structural space can be expressed by the number of descriptors that capture information about the pharmacophore pattern. Some machine learning techniques combined with weighting and selecting algorithms were applied to specify the minimal set of pharmacophoric features potentially valid for ligand-site interactions [59]. A meaningful comparison of the shape/charge intensities produced on the molecular surface and values of target affinity can be performed using comparative molecular surface analysis (CoMSA) [68]. A self-organizing network (SOM) is composed of a single layer of neurons arranged in a 2D plane with well-defined topology [69]. The closely related objects are located in the proximal neurons of the square map because a neural network automatically adapts itself to the input data. In other words, a planar image of the multidimensional property space is generated, where objects located in neighboring neurons possess theoretically similar properties—most groups have a distinct location in specific regions of the map. The presence of electrostatic and/or steric features, that are potentially valid for ligand-receptor complementarity and recognition, can be detected using the recurrent variable selection method conjugated with the PLS procedure (IVE-PLS) [70]. Briefly, the whole computer implementation includes four stages: (**1**) standard PLS analysis with LOO-CV to assess the performance of the PLS model, (**2**) elimination of a matrix column with the lowest *abs(mean(b)*/*std(b))* value, (**3**) standard PLS analysis of the new matrix without the column eliminated in stage (**2**), (**4**) recurrent repetition of stages (**1**)–(**3**) to maximize the LOO parameter. In order to estimate the model robustness and SAR predictive power, the multiple interchangeable division of the dataset into training/test subsets was applied. The descriptor-driven consensus pharmacophore pattern is derived on the basis of the validated models; however, our models are not expected to be predictive externally—extrapolation might be elusive [71]. The chosen training/test samplings within highly populated areas of $q_{cv}^{2}$ > 0.5 versus $q_{test}^{2}\;>\;0.5$ performance were selected to produce ‘an average’ pharmacophore. On the contrary to the standard procedure for a single training/test subset, an attempt was taken to specify a common ensemble of variables that contribute (un)-favorably to the observed activity simultaneously in all chosen models. Thus, the columns annotated with the highest stability *abs*(*mean*(*b*)/*std*(*b*)) for each of the chosen models were identified using the IVE-PLS methodology. The cumulative sum of common columns for the selected models was specified and normalized to the range of [0–1]. The relative contribution of each variable is weighted by the magnitude and sign of the corresponding regression coefficient; therefore, color code signs of the descriptor affect/influence compound potency. The sign of influence is color-coded depicting not only the regions with a positive and/or negative activity contribution, but also four possible combinations of a mean charge/correlation coefficient, respectively. A simplified visual inspection of the generated pharmacophore pattern gives a clear 3D picture of the regions that should be modified to modulate the biological activity [59].

## 4. Conclusions

A series of twenty-two novel multi-target *N*-(disubstituted-phenyl)-3-hydroxynaphthalene-2-carboxamide derivatives were synthesized and characterized by the set of in vitro potency profiles. *N*-[3,5-bis(trifluoromethyl)phenyl]-3-hydroxynaphthalene-2-carboxamide (**13**) and *N*-[2-chloro-5-(trifluoromethyl)phenyl]-3-hydroxynaphthalene-2-carboxamide (**20**) demonstrated high activity against methicillin-resistant *S. aureus* isolates in the range of MICs from 0.16 to 0.68 µM. Compound **13** and *N*-[4-bromo-3-(trifluoromethyl)phenyl]-3-hydroxynaphthalene-2-carboxamide (**16**) showed activity against *M. tuberculosis* (both MICs approx. 10 µM) comparable with rifampicin. Synergistic in vitro studies showed an increase in activity of ciprofloxacin in combinations with compound **16** or *N*-(4-bromo-3-fluorophenyl)-3-hydroxynaphthalene-2-carboxamides (**17**) against MRSA SA 630 isolate, which could be related to the inhibition of efflux pumps. The similarity-related property space assessment for the congeneric series of structurally related carboxamide derivatives was performed using the principal component analysis. It is noticeable that very active *N*-[3,5-bis(trifluoromethyl)phenyl]-3-hydroxynaphthalene-2-carboxamide (**13**) occupies a distinct location of the PC1 vs. PC2 plane, while fairly potent compounds are grouped together. Interestingly, different distribution of mono-halogenated carboxamide derivatives with the –CF_3_ substituent (compounds **15**, **16**, **18**–**22**) is accompanied by the increased activity profile. Moreover, the quantitative sampling of similarity-related activity landscape provided a subtle picture of favorable and disallowed structural modifications that are valid for determining the activity cliffs. A symmetric matrix of Tanimoto coefficients indicated the structural dissimilarities of dichloro-substituted (compounds **9**–**12**) and dimetoxy-substituted (compounds **1**, **2**) isomers from the remaining ones. The potent compound **13** (R = 3,5-CF_3_) is accompanied by inactive 3-hydroxy-*N*-[4-methyl-3-(trifluoromethyl)phenyl]naphthalene-2-carboxamide (**14**) where one trifluoromethyl group was isomerically (*meta*→*orto*) replaced with one methyl substituent. It seems that further dense sampling of rough regions (the upper right corner) in the numerical SALI plane (T > 0.75 and pIC_50_ > 2) is necessary to specify valid SAR boundaries for the investigated series of carboxamides. An advanced method of neural network quantitative SAR was engaged to illustrate the key 3D steric/electronic/lipophilic features of the ligand-site composition by the systematic probing of the functional group. On the other hand, it cannot be expected that even robust and validated models can reliably predict the modeled property for the entire universe of chemicals; therefore, the application boundary should be investigated using similarity concept and biological surface analysis. From a philosophical point of view, it is impossible to specify an absolute measure of predictivity, as it considerably depends on the selection of datasets and the statistical approach that is engaged, but the great advantage of the QSAR paradigm lies not in the extrapolation, as was stated by Hansch [14]. Combining molecular similarity concept with hypothesis-based pharmacophore mapping may enhance the probability of finding potent compounds in drug discovery projects.

## Figures and Tables

**Table 1:** Compositions of *N*-disubstituted 3-hydroxynaphthalene-2-carboxanilides, in vitro antistaphylococcal activities MIC [μM] compared to ampicillin (AMP) and ciprofloxacin (CPX), in vitro antitubercular activity MIC [μM] compared to isoniazid (INH) and rifampicin (RIF).

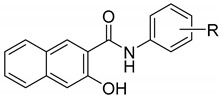						
No.	R	MIC [μM]				
		SA	MRSA 63718	MRSA SA 630	MRSA SA 3202	MT
**1.**	2,5-OCH_3_	198	792	792	792	792
**2.**	3,5-OCH_3_	198	792	792	792	792
**3.**	2,5-CH_3_	879	879	879	879	256
**4.**	2,6-CH_3_	27.4	54.9	27.4	27.4	54.9
**5.**	3,5-CH_3_	879	879	879	879	110
**6.**	2,5-F	856	856	856	856	879
**7.**	2,6-F	26.7	856	53.4	107	107
**8.**	3,5-F	856	856	214	856	53.4
**9.**	2,5-Cl	770	770	770	770	770
**10.**	2,6-Cl	193	385	385	385	385
**11.**	3,4-Cl	770	770	770	770	770
**12.**	3,5-Cl	770	770	770	770	12.0
**13.**	3,5-CF_3_	0.158	0.626	0.158	0.626	10.0
**14.**	3-CF_3_-4-CH_3_	741	371	741	371	741
**15.**	3-CF_3_-4-F	11.4	11.4	11.4	11.4	22.9
**16.**	3-CF_3_-4-Br	2.43	4.78	4.78	4.78	9.75
**17.**	3-F-4-Br	5.54	5.54	5.54	5.54	11.1
**18.**	5-CF_3_-2-F	11.4	11.4	11.4	11.4	11.4
**19.**	5-CF_3_-3-F	11.4	11.4	11.4	11.4	11.4
**20.**	5-CF_3_-2-Cl	0.342	0.684	0.342	0.342	10.9
**21.**	4-CF_3_-3-F	5.72	5.72	5.72	5.72	11.4
**22.**	4-CF_3_-2-Br	624	624	624	624	624
**AMP**	**–**	5.72	45.8	45.8	45.8	–
**CPX**	**–**	0.751	48.3	48.3	48.3	–
**INH**	**–**	**–**	**–**	**–**	**–**	36.5
**RIF**	**–**	**–**	**–**	**–**	**–**	9.72

SA = *Staphylococcus aureus* ATCC 29213; MRSA = clinical isolates of methicillin-resistant *S. aureus* 63718, SA 630, and SA 3202 (National Institute of Public Health, Prague, Czech Republic); MT = *Mycobacterium tuberculosis* H37Ra ATCC 25177.

**Table 2:** Effect of tested compounds in combination with ciprofloxacin (CPX) and oxacillin (OXA) against isolate MRSA SA 630. MICs (μg/mL) of compounds alone and observed in the synergy experiment are shown in parentheses. In the case of additivity and synergy, concentrations of tested compound/CPX (µg/mL) providing this effect are shown.

Combination (MIC [μg/mL])	FIC Index	Comb. Effect (MICs [μg/mL])
Comp. **13** + CPX (0.125/128)	1.000	IND
Comp. **16** + CPX (8/128)	0.375	SYN 1/32
Comp. **17** + CPX (1/128)	0.500	SYN 0.25/32
Comp. **20** + CPX (0,5/128)	1.004	IND
Comp. **21** + CPX (0.125/128)	0.75	ADD 0.0625/32; 0.03125/64
Comp. **13** + OXA (0.125/64)	1.004	IND
Comp. **16** + OXA (8/64)	1.000	IND
Comp. **17** + OXA (1/64)	1.125	IND
Comp. **20** + OXA (0,5/64)	1.002	IND
Comp. **21** + OXA (0,125/64)	1.125	IND

FIC = fractional inhibitory concentration; IND = indifference; ADD = additivity; SYN = synergy.
